# Requirements for occupational exposure limits in psychosocial risk assessment: What we know, what we don’t know and what we can learn from other disciplines

**DOI:** 10.5271/sjweh.4247

**Published:** 2025-11-01

**Authors:** Roman Pauli, Jessica Lang, Andreas Müller, Yacine Taibi, Thomas Kraus, Yannick Metzler

**Affiliations:** 1 Institute for Occupational, Social and Environmental Medicine, RWTH Aachen University, Aachen, Germany.; 2 Department of Work and Organizational Psychology, University of Duisburg-Essen, Duisburg, Germany.; 3 Occupational Health Management, Thyssenkrupp Steel Europe AG, Duisburg, Germany.; 4 Leibniz Research Centre for Working Environment and Human Factors, Dortmund, Germany.

**Keywords:** cumulative exposure, dose–response relationship, health-based threshold value, psychosocial hazard

## Abstract

**Objectives:**

This discussion paper aims to provide recommendations for the development of occupational exposure limits (OEL) for psychosocial hazards. By comparing the characteristics of non-psychosocial and psychosocial hazards at work as well as approaches to derive occupational limit values for both types of hazards, the paper summarizes conceptual requirements and methodological perspectives for OEL in psychosocial risk assessment.

**Methods:**

An interdisciplinary working group comprised of academics, active practitioners in company occupational health management and members of national committees advising policymakers conducted regular face-to-face and online meetings between October 2022 and August 2024 to draft a narrative review and discussion of the current state of research on OEL for psychosocial hazards within the fields of psychology, sociology and medicine.

**Results:**

The current field of research is in its early stages, indicated by individual efforts and a lack of joint decision-making. Existing approaches towards OEL focus on disease-level outcomes (eg, burnout, depression), which limits their effectiveness for primary prevention and identifying early warning signs of harm.

**Conclusion:**

Based on the limited existing literature, we recommend (i) the use of outcome variables that enable detection of early stages of adverse effects aligned with the no-observed adverse effect level (NOAEL) and the lowest-observed-adverse effect level (LOAEL), (ii) standardization and harmonization of hitherto independent assessments of identical hazards, and (iii) policy-level actions to foster collaborative decision-making based on the full spectrum of scientific evidence.

With the rising prevalence of mental health disorders, psychosocial occupational hazards have become a major public health concern that calls for urgent action (1). Occupational hazards refer to any condition, agent or activity that potentially causes harm at a specific level of risk (2). In the European Social Charter, the contracting parties declared one of their policy objectives to be to secure the right of all workers to safe and healthy working conditions. The EU Framework Directive on Safety and Health at Work (89/391/EEC), which requires employers to identify and combat risks to the health and safety of employees, was an important step towards this goal. Most European member states added country-specific regulations explicitly addressing psychosocial hazards at work (3). In addition, efforts for advancing psychosocial risk management have been taken, for example in the European Psychosocial Risk Management Framework (PRIMA-EF) or standards for psychosocial risk management (4, 5).

In contrast to these efforts stands the considerable lack of practical implementation of psychosocial risk assessments in EU organizations: >70% of EU companies have not implemented an assessment of psychosocial risks in their occupational health and safety management, mostly due to a lack of knowledge, fear of potential conflict and ambiguous responsibilities (6, 7). A key challenge is determining the exposure level at which psychosocial hazards harm employee health and identifying the appropriate threshold for occupational health and safety practices (8, 9). Criteria for evaluating the risk probability of psychosocial hazards at work, such as occupational exposure limits (OEL) established for chemical agents, have long been called for (10). A systematic conceptualization of the requirements of such OEL, however, is still pending. This raises the question to what extent established approaches in evaluating non-psychosocial hazards can be transferred to psychosocial risk assessment to overcome these shortcomings.

Therefore, this discussion paper (i) critically discusses concepts and current practices in developing OEL occupational exposure limits for non-psychosocial and psychosocial hazards, (ii) characterizes non-psychosocial and psychosocial hazards by their supposed differences, and (iii) derives conceptual and methodological recommendations for the development of OEL for psychosocial hazards at work. With that, the present contribution provides a conceptual framework of requirements for OEL in psychosocial risk assessment that serves as a roadmap for research on the impact of psychosocial hazards on early health changes and their application in legal policies and procedures.

## Occupational exposure limits for psychosocial hazards – current state of the art

The aim of any risk assessment is to evaluate if exposure to a hazard may pose a risk to health and well-being, and thus to determine when occupational health and safety measures are required. Three suggested approaches to address this issue are empirical comparative values, discussions in joint workshops, or data-driven approaches towards limit values (8).

Empirical comparative values [eg, from job-exposure matrices (11, 12)] compare estimated exposure levels for hazards across occupations as averages of an as-is state within occupations. Comparative values provide a reference point for organizations to contextualize their own data, identify relative areas of concern, and prioritize action where resources are limited. However, they are not designed to protect individual employees from overexposure to workplace hazards as they do not reflect health-based thresholds. This approach is furthermore insensitive to hazards that are already critical in the benchmark group and may therefore underestimate workplace hazards: If a group’s working conditions are equally poor as is the industry’s standard, empirical comparative values are insufficient to identify occupational health and safety requirements. Discussions in joint workshops, on the other hand, enable participation of various stakeholders, including employees, in the interpretation of the results and thus offer opportunities to derive customized and accepted measures (13). Depending on the composition of the group, however, the evaluation of hazards can be biased by subjective or interest-based assessments (14) when, for example, employers and employees differ in their assessments of the relevance of different hazards.

To overcome these shortcomings, approaches towards limit values based on health-related criteria have been suggested: These approaches use binary classifiers derived, eg, from clinical indicators of depression (9, 15, 16) or burnout (17) to differentiate healthy from ill employees. Other than comparative values, limit values provide absolute estimates for psychosocial hazards and are less biased by subjective judgments or interest-driven assessments compared to discussions in joint workshops. On the other hand, it is debatable whether a value derived from the optimal trade-off between sensitivity and specificity represents a meaningful threshold, even when the association between the binary criterion and the indicator is weak. Still, these methodological approaches paved the way towards OEL for psychosocial hazards. However, the current state of research also reveals important unresolved issues that upcoming research in this area will have to address. Before discussing four essentials to be considered for OEL for psychosocial hazards, we briefly introduce important terms and concepts from research on OEL for non-psychosocial hazards.

## Occupational exposure limits for non-psychosocial hazards – a blueprint?

Initially adopted to reconcile existing guidelines on exposure to airborne workplace chemicals (18), OEL were conceived as time-weighted average concentrations of a substance at the workplace, below which acute or chronic damage to the health of employees is not to be expected. Today, regulatory authorities, federal agencies or expert committees at national and international level have established OEL for many chemical agents (19) as well as ionizing radiation, electromagnetic fields, artificial optical radiation, and noise (20). Exposure duration usually refers to an 8-hour time-weighted average or a 15-minute short-term exposure limit based on a 40-hour work week. Short-term exposure limits are applied when health effects are expected from single or peak events (eg, in noise), whereas time-weighted averages are applied under continuous or repeated exposure. The common approach to determine exposure limits encompasses a comprehensive review of animal or human studies, epidemiological data and other sources, to assess the empirical evidence for adverse health effects (21, 22). Therefore, OEL do not result from one single study but are agreed upon in discussions involving multiple stakeholders. Two important concepts in this discussion are the no-observed-adverse-effect level (NOAEL) (23) and the lowest-observed-adverse-effect level (LOAEF) (24), both of which are used to establish safe exposure levels for hazardous substances. NOAEL and LOAEL are derived from healthy working populations or extrapolated from experimental data and should be established whenever possible based on available evidence (25).

## Supposed differences between non-psychosocial and psychosocial hazards

The extent to which established procedures for determining OEL for non-psychosocial risks can be transferred to psychosocial risks is controversial (26). Central to this discussion are key differences in the materiality of hazards, mechanisms involved, dose–response relationships, and the contextual nature of effects. By closely examining these dimensions, we question the relevance of supposed differences and advocate for incorporating insights from non-psychosocial risk models into psychosocial risk assessments.

## (Im-)materiality of hazards

Non-psychosocial hazards are mostly described in terms of their material properties, ie, their molecular composition, constitutional state or other characteristics that can be identified via highly standardized measurement procedures. Psychosocial hazards are considered from two complimentary perspectives: The first perspective assumes psychosocial hazards as objective entities with associated health effects, independent of an individual employee’s constitution or subjective interpretation (14). Accordingly, this perspective calls for an exposure assessment of psychosocial hazards that is as unbiased as possible by characteristics of the individual as well as for occupational limit values independent of individual appraisal (27, 28). The second perspective emphasizes workers subjective and collective appraisals and coping mechanism (29, 30). This constructivist perspective highlights both idiosyncratic and social processes to explain health effects of psychosocial hazards (31), but challenges the reliability and validity of measurements: The two most frequently used methods in psychosocial risk assessment, standardized employee self-reports and observer ratings by experts, are both subject to human perception and processing and, therefore, contaminated with varying levels of between-person variation. Therefore, findings on the correlation between self-reported and observer-rated job demands are mixed (14, 32). While employee surveys of working conditions are a central element for assessing mental stress, methodological research into decreasing the idiosyncratic share of these assessments is still relatively young (27). Moreover, psychosocial risk assessments frequently omit an assessment of individual employee characteristics in order to emphasize focus on working conditions rather than personal traits. This, however, limits scientific analysis by omitting valuable information about causes of inter-individual differences in the perception and evaluation of psychosocial hazards (eg, personality differences) as well as opportunities for (statistical) control of these differences in order to enhance the validity of the results.

## Multiplicity of mechanisms

The relationship between non-psychosocial hazards and health impairments is thought to be explained by rather unambiguous physiological pathways. Examples are high noise levels that lead to hearing loss by damaged hair cells in the ear or unprotected contact with acids causing severe tissue damages by denaturing the proteins in skin cell membranes. Psychosocial hazards, on the other hand, are assumed to cause or contribute to multiple health impairments through multiple physiological (eg, metabolic or cardiovascular reactions), behavioral (eg, maladaptive coping styles) and cognitive pathways (eg, negative appraisals). For example, high job strain (eg, low job control coupled with high job demands) has been identified a risk factor for coronary heart diseases, obesity, as well as depression (33). However, the unambiguous assignment of pathways from non-psychosocial hazards to diseases is oversimplified as the expected health effects following exposure to non-psychological hazards cannot be predicted without ambiguity. For example, a biological limit value for acrylamide – a chemical stabilizer used as grouting agent in tunnel building – was established based on studies where workers showed neurotoxic symptoms (34). However, different limit values were derived for its carcinogenic effects (35). Therefore, different limits are applied depending on the respective outcome. Such findings resulted in the concept of ‘uncertainty factors’ in risk assessment that acknowledge variations in health effects caused by exposure to hazards across specifically vulnerable subgroups (36). Exposure limits for psychosocial hazards should likely be established despite varied mechanisms. Multiple limits might be needed for different outcomes or occupational groups, with the lowest limit preventing the least serious impairment being applicable in practice.

## Dose–response relations and exposure duration

For most non-psychosocial hazards, dose–response relations are often falsely reduced to oversimplified linear associations. A dose–response relationship describes the magnitude of the response as a non-linear function of exposure, also including factors like time or severity. Research on psychosocial hazards has predominantly focused on linear relationships, with some evidence for non-linear associations (37). To derive meaningful dose–response curves, information is needed on confounding variables, vulnerability, prioritizing critical outcomes of interest, and exposure duration. Statistical considerations for estimating threshold values for exposure to working conditions have been discussed elsewhere (38, 39). A crucial aspect in this regard is that psychosocial hazards are mostly evaluated from an epidemiological point of view and lack an assessment of exposure duration. However, short-term effects of psychosocial hazards from peak events, such as exposure to violence, are well documented (40) just as long-term exposure effects are (33). For psychosocial hazards, the consideration of cumulative effects of repeated exposure over short (41, 42) and long (43) periods of time is still relatively young. The required continuous measurement of psychosocial stress and individual strain poses significant challenges for research designs. However, the widespread presence of sensor technology in modern end-user devices such as smartphones and smartwatches (44), combined with innovative approaches to accessing data collected by individuals (45), opens up new potentials for workplace (mental) health research.

## Contextuality of effects

At first glance, health effects of non-psychosocial hazards seem straight forward. High asbestos dust exposure is likely to damage the respiratory system of an unprotected individual; however, contextual factors (eg, smoking habits) moderate this risk (46). Likewise, the impact of psychosocial hazards can vary by context, shaped by subjective perceptions, occupational roles, workplace social structure, coping mechanisms, individual susceptibility, or the complex interplay of multiple psychosocial factors. However, there is debate over interaction hypotheses (47, 48). Exposure limits may hence differ across occupational groups and tasks (49) or attributes of the social context (50).

In short, differences between non-psychosocial and psychosocial hazards may only marginally affect the way in which the risk of hazards at work is conceptualized for employee health. Rather, both types of hazards are associated with similar challenges for determining cause-and-effect and dose–response relationships, which are crucial components of discussions around occupational limit values. Therefore, a close look at established approaches to determining OEL for non-psychosocial hazards can provide a valuable contribution to the development of the same for psychosocial hazards.

## Four essentials for occupational exposure limits for psychosocial hazards

While there are well-established concepts and approaches for setting exposure limits in biological, chemical, and ergonomic hazards, research on limit-values for psychosocial hazards is in its early stages. At the same time, evidence for substantial differences between non-psychosocial and psychosocial hazards at work is limited. Recent acknowledgements of epigenetic differences in reactions to stress (51) as well as of methodological improvements to control for inter-individual differences in psychosocial risk assessment (27) indicate growing efforts to better understand the mechanisms that lead to varying risk potentials of hazards at work. Based on this, we derive the following essentials to be addressed by future research efforts to determine OEL for psychosocial hazards at work:

*1. Assess associations with short-term changes in psychophysiological reactions.* The majority of current research on psychosocial hazards is based on data from surveys that had been originally developed for purposes other than determining OEL. Frequently, limit values result from secondary analyses of data, eg, epidemiological studies or psychosocial risk assessments with a primary focus on severe health outcomes like disorders of the musculoskeletal systems, burnout or depression. However, the parameters conventionally used to indicate psychological hazards in such study designs address health outcomes at a stage that is too late for early detection of symptoms and primary prevention, ie, actions and strategies designed to prevent the onset of a health condition or disease before it occurs. A threshold derived from an actual disease overlooks the health risks emerging from early stages of elevated psychosocial hazards at work. While the pathogenic effect of excessive psychosocial demands is well documented, the thresholds beyond which psychosocial hazards *start* to impact employees’ health are unknown. We therefore strongly advise future research to investigate NOAEL and LOAEL as early indicators of adverse (mental) health effects rather than investigating pathological states (mental disorders) resulting from exposure to psychosocial hazards.

We therefore invite researchers to shift their attention towards processes of change (52), eg, short-term changes in psychophysiological reactions that might even be reversible after exposure and, therefore, are below the threshold of actual health impairments. Examples include the point at which no significant decline in eg, energetic arousal, cognitive function or self-reported mood is observed. In addition, the LOAEL for psychosocial hazards might be used to identify situations at which stress-related symptoms (eg, fatigue, irritability, rumination) *start* to emerge. LOAEL help to identify early signs of strain and therefore seem conceptually meaningful rather than merely statistically plausible limit values to prompt preventive action. As a theoretical framework, eg, the Allostatic Load Model (53) describes long-term effects of the physiological response to stress, where multiple systems (autonomic nervous system, hypothalamic–pituitary–adrenal axis, cardiovascular, metabolic, and immune systems) interact to protect the body when responding to external stress. If we set LOAEL to the initial occurrence of physiological reactions to psychosocial hazards, psychosocial risks can be derived from an allostatic load index of the physiological response like elevation of stress hormones, heart rate or hypermetabolism (54). Furthermore, we need a better understanding of the exposure at which no adverse effects can be observed among employees, ie, working conditions that can be considered safe or acceptable. In this regard, NOAEL could serve as practical guidelines for occupational health and safety, indicating thresholds below which implementation of protective measures is not mandatory. Beyond initial efforts to identify exposure levels associated with adverse health outcomes (9), to the best of our knowledge, the concept of NOAEL still represents the “what we don’t know” in current research on psychosocial hazards.

From the authors’ point of view, such comprehensive knowledge is a prerequisite to be able to classify insights from more complex analyses, eg, of interaction effects of different hazards. While traditional theoretical models in work and organizational psychology account for such complex interactions, their strong focus on employees’ perceptions of demands is a potential bias of the associations between stress and strain. This implies that – as a result of stressor-strain analyses – meaningful limit values rely on an unbiased assessment of hazards. Therefore, work on the methodological effects in psychosocial risk assessment is an important avenue for future research.

*2. Be precise about the relationship between cause/dose and effect.* From the authors’ perspective, the information already available from survey research is not sufficiently exploited in a way that allows for insights into the specific cause–effect as well as dose–response relationships between psychosocial hazards and health outcomes. An example is the frequently used approach of transforming continuous outcome variables into dichotomous categories. To predict the probability of an undesired outcome from logistic regression models and evaluate true-positive and false-positive classifications of such outcomes via ROC curves, outcome variables initially collected on continuous scales are converted into dichotomous categories of healthy and sick employees. However, this approach assumes that, for instance, individuals just below a given cut-off value are ill and individuals just above it are healthy – even though the differences between healthy and ill individuals close to the cut-off value is negligible compared to the differences among individuals within the groups of healthy and sick individuals. While outcomes can be dichotomous (eg, diagnosed versus no diagnosed disease), ordinal (eg, number of sickness absences) as well as continuous (eg, well-being), dichotomizing continuous outcomes creates artificial distinctions that do not reflect the true nature of the effects and can lead to unreliable conclusions about the actual risk potential of hazards. We recommend future research makes use of the full range of information in the data, leading to accurate information about dose–response relationships between varying levels of hazards and health outcomes. A statistical method for assessing thresholds in epidemiological studies has been suggested in (39). In doing so, the temporal trajectories of the exposure need to be addressed to detect and differentiate subacute from sub chronic, chronic and lifetime effects (21). Therefore, experimental studies in controlled environments may offer insights into the psychobiological mechanisms of stressor-strain associations, complementing findings from survey-based studies and addressing concerns such as the single source bias in job stress research (55). However, long-term, real-world occupational settings challenge the ecological validity of experimental setups, while at the same time realistic laboratory exposure manipulations may raise ethical concerns. Therefore, epidemiological studies with shortened assessment intervals (56) and harmonized measures across studies could enhance causal inference while maintaining ecological validity. This way, processes of subjective appraisal could be explicitly modeled to understand their share in substantial variance components (32). All these different methodological approaches come with their own advantages and disadvantages. Only by putting together individual pieces to the puzzle, research can move forward in expanding the causal mechanisms behind exposure to workplace hazards and employee health (57).

*3. Provide generalizable OEL for psychosocial hazards.* As of now, OEL for psychosocial hazards have been derived as cut-off values for the very survey instruments with which the hazards had been assessed. At the same time, there is a lack of exchange about the similarities and differences between (i) different measurement methods and instruments and (ii) the conceptualization of latent variables underlying these measurements. Despite initial suggestions for taxonomies of work stressors [eg (58),], a systematic discussion of the generalizability of the limit values collected this way across different measurement methods is still pending. In order to compare latent constructs – such as psychosocial hazards – survey research has developed ‘data harmonization’ or ‘integrative data analysis’ (59), (60), that can be utilized to overcome the current disintegration of approaches. The aim of these methods is to convert different scalings or wordings across surveys into a common metric to allow for comparisons across surveys. Depending on the distribution characteristics of the data and the goal of the harmonization procedure, different data transformation processes, ie, data linking and linear or equipercentile equating, are used (61). These methods can help to move the discussion about psychosocial hazards away from the individual survey instruments towards the latent constructs underlying the assessments. In addition, although mandated by law, psychosocial risk assessment currently is an unregulated market with an increasing amount of service providers offering self-developed questionnaires with sometimes questionable psychometric properties for commercial use. Therefore, additional knowledge and guidelines for reliable and valid psychosocial risk assessment is required.

Finally, there is a lack of knowledge about the generalizability of limit values determined across different groups of activities and individuals. The multiplicity of factors influencing the effect of psychosocial hazards, including individual differences, subjective interpretations, and contextual elements, complicates the development of a standardized assessment methodology. Future research must accordingly address the question for which specific groups of employees the particular limit values may apply to. This may result in (i) limit values set at a level low enough to prevent harm for all occupational groups, (ii) an examination of individual differences with limit values specific to occupational groups or (iii) the acknowledgement that, for some psychosocial hazards, there must be zero tolerance of exposure (eg, bullying, sexual harassment, violence at work).

*4. Institutionalize discussion about suitable OEL.* Establishing OEL for psychosocial hazards entails both empirical research and negotiations over acceptable risks (21). In addition, decisions about occupational safety measures as a reaction to psychosocial hazards at work results from negotiation processes that must balance the interests of employers and employees. Therefore, we consider the current practice of suggesting OEL for a number of psychosocial hazards measured with one survey instrument in a single study to be a methodological problem. The development of OEL for hazardous substances is a result of agreement processes based on the current state of knowledge about eg, the toxicity of a given substance by considering *all* available results from animal, epidemiological and human exposure studies. We suggest future endeavors on OEL for psychosocial hazards take this approach as a blueprint to summarize the findings gained from the entire spectrum of methods from experimental, observational, epidemiological studies as well as qualitative and quantitative survey results. We therefore encourage establishing forums for exchange and discussion of these findings and joint decision-making about the scientific validity and practical usefulness of OEL for psychosocial risk assessment.

## Future research outlook: evolution of occupational exposure limits for psychosocial hazards

**Figure 1:**
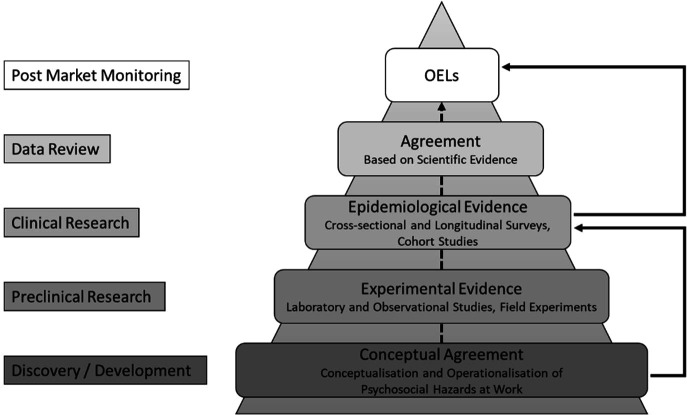
Ideal (dotted lines) versus actual (solid lines) evolution of occupational exposure limits (OELs) for psychosocial hazards at work compared to the drug development process. Adapted from (11).

In the light of these findings, the current state of research on OEL for psychosocial hazards appears incomplete and fragmented. In figure 1, we use the process of drug development as an analogy to compare an ideal evolution of the development of limit values for psychosocial hazards with the actual current practice:

At different stages, this process involves various stakeholders from scientists (in the experimental and epidemiological phases) to occupational health and safety authorities in agreements and policy developmental phases. Equal to drug development, limit values for psychosocial hazards should start with a developmental step to agree on conceptual definitions. These concepts can then be validated in the preclinical or experimental step by investigating associations with short-term changes in psychophysiological reactions, eg, with laboratory and observational studies. Should this step yield a proof of concept, it is then time to move on to the applied research in companies as an epidemiological step to gather, eg, survey data of varying intensity of temporal trajectories of associations between hazards and preclinical stress-related symptoms rather than clinical mental conditions. In the data review or agreement step, all findings from the previous steps are considered in an agreement process resulting in recommendations for OEL. A final monitoring phase is used to assess the long-term outcomes of the entire process. In contrast to this ideal evolution, the black arrows indicate the actual current field of research that jumped from an informal agreement about different categories of psychosocial hazards to (mainly cross-sectional) epidemiological evidence, which in turn directly resulted in suggestions for limt values. OEL are based on the fundamental assumption that exposure to certain workplace conditions leads to mental health conditions. While this (statistical) association—particularly for depression—is well supported by existing epidemiological evidence, there remains a lack of theoretical understanding regarding the underlying mechanisms (62). In order to close these gaps, we conclude there is an urgent need to foster the harmonization of – as of now uncoordinated – research efforts to determine limit values for psychosocial hazards at work. Based on earlier recommendations of Deveau et al (19), in table 1 we propose a stepwise catalogue of requirements, studies on this matter should address in order to foster coordinated efforts as well as to provide transparent documentation and standardized reporting.

**Table 1:** Recommended stepwise approach towards limit values for psychosocial hazards at work. [NOAEL=no-observed-adverse-effect level; LOAEL=lowest-observed-adverse-effect level]. Based on Deveau et al (19).

1.	Define the psychosocial hazard of interest and develop the problem formulation (ie, specify the targeted job population, specify the exposure duration, specify the theory to explain the effects of the hazards)
2.	Review the scientific literature guided by the problem formulation (eg, original studies, reviews, reports)
3.	Specify the operationalization of the psychosocial hazard
4.	Justify the outcome of interest and its operationalization (ie, prevention via NOAEL/LOAEL, identification of disease-like status),
5.	Follow a standardized scheme for documenting and reporting
	5.1. summarize the state-of-the-art relevant for the problem formulation
	5.2. justify methodological decisions (eg, target population, statistical procedures)
	5.3. report all relevant dose-response and dose-effect relationships
	5.4 highlight the critical effect and its consequences for employee health
6.	Submit results for review by external parties

In the history of OEL, numerous commissions, expert groups and bodies on national and international levels had been found to develop and discuss critical levels of human exposure to chemicals at work. With increasing psychosocial demands in contemporary workplaces, now is the time to develop comparable regulatory bodies or consensus groups to tackle work‐related psychosocial hazards. In this paper, we have outlined the research gaps that need to be closed to develop a comprehensive approach to the topic. Researchers can close these gaps by applying an expanded range of methodological approaches. At the policy-level, collaboration among research groups should be facilitated, encouraging dialogue and informed decision-making based on emerging research findings. We believe these actions are a prerequisite for setting OEL based on the full range of scientific evidence to protect workers from psychosocial hazards at work.

## References

[1] Schulte PA, Sauter SL, Pandalai SP, Tiesman HM, Chosewood LC, Cunningham TR et al. An urgent call to address work-related psychosocial hazards and improve worker well-being. Am J Ind Med 2024 Jun;67(6):499–514. 10.1002/ajim.23583 38598122 PMC11980372

[2] Alli BO. Fundamental principles of occupational health and safety. 2^nd^ ed. Geneva: International Labour Office; 2008.

[3] Jain A, Torres LD, Teoh K, Leka S. The impact of national legislation on psychosocial risks on organisational action plans, psychosocial working conditions, and employee work-related stress in Europe. Soc Sci Med 2022 Jun;302:114987. 10.1016/j.socscimed.2022.114987 35500313

[4] Leka S, Jain A, Widerszal-Bazyl M, Żołnierczyk-Zreda D, Zwetsloot G. Developing a standard for psychosocial risk management: PAS 1010. Saf Sci 2011;49(7):1047–57. 10.1016/j.ssci.2011.02.003

[5] ISO 10075-3:2004-12, Ergonomic principles related to mental workload - Part 3: Principles and requirements concerning methods for measuring and assessing mental workload, Vol 13.180. Genf: International Organization for Standardization; 2004. Available from: https://www.austrian-standards.at/en/shop/din-en-iso-10075-3-2004-12~p3094262?toggle=1

[6] Ales E, Bell M, Deinert O, Robin-Olivier S, editors. International and European labour law: Article-by-article commentary. 1^st^ ed. International and European business law. Baden-Baden, München, Oxford: Nomos; Beck; Hart; 2018. p. 1678.

[7] Eurofound and EU-OSHA. Psychosocial risks in Europe: Prevalence and strategies for prevention. Luxembourg: Publications Office of the European Union; 2014.

[8] Metzler YA, von Groeling-Müller G, Bellingrath S. Better safe than sorry: methods for risk assessment of psychosocial hazards. Saf Sci 2019;114:122–39. 10.1016/j.ssci.2019.01.003

[9] Kyaw-Myint SM, Strazdins L, Clements M, Butterworth P, Gallagher L. A method of identifying health-based benchmarks for psychosocial risks at work: A tool for risk assessment. Saf Sci 2017;93:143–51. 10.1016/j.ssci.2016.11.016

[10] Benavides FG, Benach J, Muntaner C. Psychosocial risk factors at the workplace: is there enough evidence to establish reference values? J Epidemiol Community Health 2002 Apr;56(4):244–5. 10.1136/jech.56.4.244 11896129 PMC1732115

[11] Peters S. Although a valuable method in occupational epidemiology, job-exposure -matrices are no magic fix. Scand J Work Environ Health 2020 May;46(3):231–4. 10.5271/sjweh.3894 32356897

[12] Hanvold TN, Sterud T, Kristensen P, Mehlum IS. Mechanical and psychosocial work exposures: the construction and evaluation of a gender-specific job exposure matrix (JEM). Scand J Work Environ Health 2019 May;45(3):239–47. 10.5271/sjweh.3774 30614504

[13] Abildgaard JS, Hasson H, von Thiele Schwarz U, Løvseth LT, Ala-Laurinaho A, Nielsen K. Forms of participation: the development and application of a conceptual model of participation in work environment interventions. Econ Ind Democracy 2020;41(3):746–69. 10.1177/0143831X17743576

[14] Schneider I, Mädler M, Lang J. Comparability of self- and observer-ratings in occupational psychosocial risk assessments – Is there agreement? BioMed Res Int 2019 Jun;2019:8382160. 10.1155/2019/8382160 31309118 PMC6594325

[15] Diebig M, Angerer P. Description and application of a method to quantify criterion-related cut-off values for questionnaire-based psychosocial risk assessment. Int Arch Occup Environ Health 2021 Apr;94(3):475–85. 10.1007/s00420-020-01597-4 33140836 PMC8295087

[16] Zeike S, Ansmann L, Lindert L, Samel C, Kowalski C, Pfaff H. Identifying cut-off scores for job demands and job control in nursing professionals: a cross-sectional survey in Germany. BMJ Open 2018 Dec;8(12):e021366. 10.1136/bmjopen-2017-021366 30530574 PMC6303688

[17] Dettmers J, Stempel CR. How to Use Questionnaire Results in Psychosocial Risk Assessment: Calculating Risks for Health Impairment in Psychosocial Work Risk Assessment. Int J Environ Res Public Health 2021 Jul;18(13):7107. 10.3390/ijerph18137107 34281047 PMC8296915

[18] Paustenbach DJ, Cowan DM, Sahmel J. The History and Biological Basis of Occupational Exposure Limits for Chemical Agents. Patty’s Industrial Hygiene 2011.

[19] Deveau M, Chen C-P, Johanson G, Krewski D, Maier A, Niven KJ, et al. The Global Landscape of Occupational Exposure Limits—Implementation of Harmonization Principles to Guide Limit Selection. J Occup Environ Hyg 2015;12 Suppl 1(sup1):S127–44. 10.1080/15459624.2015.1060327. PMC4654639 26099071

[20] Kittelmann M, Adolph L, Michel A, Packroff R, Schütte M. Sommer, Sabine. Handbuch Gefährdungsbeurteilung [Risk Assessment Manual]. Dortmund; 2023.

[21] Maurer LL, Alexander MS, Bachman AN, Grimm FA, Lewis RJ, North CM et al. An interdisciplinary framework for derivation of occupational exposure limits. Front Public Health 2022 Nov;10:1038305. 10.3389/fpubh.2022.1038305 36530659 PMC9748553

[22] Wheeler MW, Park RM, Bailer AJ, Whittaker C. Historical Context and Recent Advances in Exposure-Response Estimation for Deriving Occupational Exposure Limits. J Occup Environ Hyg 2015;12 Suppl 1(sup1):S7-17. 10.1080/15459624.2015.1076934. PMC4685605 26252067

[23] Dorato MA, Engelhardt JA. The no-observed-adverse-effect-level in drug safety evaluations: use, issues, and definition(s). Regul Toxicol Pharmacol 2005 Aug;42(3):265–74. 10.1016/j.yrtph.2005.05.004 15979222

[24] Duffus JH, Nordberg M, Templeton DM. Glossary of terms used in toxicology, 2^nd^ edition (IUPAC Recommendations 2007). Pure Appl Chem 2007;79(7):1153–344.

[25] Lundberg P. National and international approaches to occupational standard setting within Europe. Appl Occup Environ Hyg 1994;9:25–7. 10.1080/1047322X.1994.10388258

[26] Rick J, Briner RB. Psychosocial risk assessment: problems and prospects. Occup Med (Lond) 2000 Jul;50(5):310–4. 10.1093/occmed/50.5.310 10975126

[27] Pauli R, Lang J. Survey Design Moderates Negativity Bias but not Positivity Bias in Self-Reported Job Stress. Eur J Psychol Assess 2024;•••: 10.1027/1015-5759/a000806

[28] Mustapha V, Rau R. Kriteriumsbezogene Cut-Off-Werte für Tätigkeitsspielraum und Arbeitsintensität: Eine Bestimmung und Evaluation [Criterion-Related Cut-Off Values for Decision Latitude and Work Intensity: Determination and Evaluation]. Diagnostica 2019;65(3):179–90. 10.1026/0012-1924/a000226

[29] Lazarus RS, Folkman S. Stress, Appraisal, and Coping: Springer Publishing Company; 1984.

[30] Levi L. Work, worker and wellbeing: an overview. Work Stress 1994;8(2):79–83. 10.1080/02678379408259981

[31] Semmer NK, Zapf D. Psychische Belastung und Beanspruchung: Die Bedeutung der Valenz und der sozialen Realität. Anmerkungen zu Ferreira und Vogt (2021) [Mental stress and strain: The importance of valence and social reality. Comments on Ferreira and Vogt (2021)]. Z Arbeitswiss 2022;76(3):375–84. 10.1007/s41449-022-00321-x 35789775 PMC9243850

[32] Semmer N, McGrath J, Beehr T. Conceptual Issues in Research on Stress and Health. In: Cooper C, editor. Handbook of Stress Medicine and Health, Second Edition: CRC Press; 2004. p. 1–43.

[33] Niedhammer I, Bertrais S, Witt K. Psychosocial work exposures and health outcomes: a meta-review of 72 literature reviews with meta-analysis. Scand J Work Environ Health 2021 Oct;47(7):489–508. 10.5271/sjweh.3968 34042163 PMC8504166

[34] Hagmar L, Törnqvist M, Nordander C, Rosén I, Bruze M, Kautiainen A et al. Health effects of occupational exposure to acrylamide using hemoglobin adducts as biomarkers of internal dose. Scand J Work Environ Health 2001 Aug;27(4):219–26. 10.5271/sjweh.608 11560335

[35] Acrylamide [MAK Value Documentation, 1992]. In: The MAK‐Collection for Occupational Health and Safety: Wiley; 2002. p. 12–21.

[36] Dankovic DA, Naumann BD, Maier A, Dourson ML, Levy LS. The Scientific Basis of Uncertainty Factors Used in Setting Occupational Exposure Limits. J Occup Environ Hyg 2015;12 Suppl 1(sup1):S55–68. 10.1080/15459624.2015.1060325. PMC4643360 26097979

[37] Pindek S, Shen W, Gray CE, Spector PE. Clarifying the inconsistently observed curvilinear relationship between workload and employee attitudes and mental well-being. Work Stress 2023;37(2):195–221. 10.1080/02678373.2022.2120562

[38] Möhner M, Nowak D. Estimation of an Exposure Threshold Value for Compensation of Silica-Induced COPD Based on Longitudinal Changes in Pulmonary Function. Int J Environ Res Public Health 2020 Dec;17(23):9040. 10.3390/ijerph17239040 33291582 PMC7729997

[39] Ulm K. A statistical method for assessing a threshold in epidemiological studies. Stat Med 1991 Mar;10(3):341–9. 10.1002/sim.4780100306 2028118

[40] Picker-Roesch C, Pauli R, Lang J. Aggression und Gewalt am Arbeitsplatz – Der Einfluss von Tätigkeitsgruppen auf die Exposition und die psychische Gesundheit der Beschäftigten [Aggression and violence in the workplace - The influence of occupational groups on the exposure and mental health of employees]. ASU 2024;59(12):764–72. 10.17147/asu-1-405964

[41] Schilling OK, Gerstorf D, Lücke AJ, Katzorreck M, Wahl HW, Diehl M et al. Emotional reactivity to daily stressors: does stressor pile-up within a day matter for young-old and very old adults? Psychol Aging 2022 Mar;37(2):149–62. 10.1037/pag0000667 34968103 PMC10074401

[42] Schilling OK, Diehl M. Reactivity to stressor pile-up in adulthood: effects on daily negative and positive affect. Psychol Aging 2014 Mar;29(1):72–83. 10.1037/a0035500 24660797 PMC6910244

[43] LaMontagne AD, Too LS, Punnett L, Milner AJ. Changes in Job Security and Mental Health: An Analysis of 14 Annual Waves of an Australian Working-Population Panel Survey. Am J Epidemiol 2021 Feb;190(2):207–15. 10.1093/aje/kwaa038 32242618

[44] Hyde JN, Runyon JR, Engineer A, Kramer B, Lindberg CM, Sternberg EM. Wearable Technologies in the Workplace: Sensing to Create Responsive Industrial and Occupational Environments Optimized for Health. In: Mehl MR, Eid M, Wrzus C, Harari GM, Ebner-Primer UW: Mobile Sensing in Psychology. Methods and Applications. Guildford Press; 2024: 542-560.

[45] Boeschoten L, Mendrik A, van der Veen E, Vloothuis J, Hu H, Voorvaart R et al. Privacy-preserving local analysis of digital trace data: A proof-of-concept. Patterns (N Y) 2022 Feb;3(3):100444. 10.1016/j.patter.2022.100444 35510190 PMC9058917

[46] Markowitz SB, Levin SM, Miller A, Morabia A. Asbestos, asbestosis, smoking, and lung cancer. New findings from the North American insulator cohort. Am J Respir Crit Care Med 2013 Jul;188(1):90–6. 10.1164/rccm.201302-0257OC 23590275

[47] Huth KB, Chung-Yan GA. Quantifying the evidence for the absence of the job demands and job control interaction on workers’ well-being: A Bayesian meta-analysis. J Appl Psychol 2023 Jun;108(6):1060–72. 10.1037/apl0001066 36442031

[48] Schaufeli WB, Taris TW. A Critical Review of the Job Demands-Resources Model: Implications for Improving Work and Health. In: Bauer GF, Hämmig O, editors. Bridging Occupational, Organizational and Public Health. Dordrecht: Springer Netherlands; 2014. p. 43–68.

[49] Pousette A, Hanse JJ. Job characteristics as predictors of ill-health and sickness absenteeism in different occupational types - a multigroup structural equation modelling approach. Work Stress 2002;16(3):229–50. 10.1080/02678370210162737

[50] Pauli R, Lang J. Collective resources for individual recovery: the moderating role of social climate on the relationship between job stressors and work-related rumination – A multilevel approach. Ger J Hum Resour Man 2021;35(2):152–75. 10.1177/23970022211002361

[51] Bernad BC, Tomescu MC, Anghel T, Lungeanu D, Enătescu V, Bernad ES et al. Epigenetic and Coping Mechanisms of Stress in Affective Disorders: A Scoping Review. Medicina (Kaunas) 2024 Apr;60(5):709. 10.3390/medicina60050709 38792892 PMC11122772

[52] Boot CR, Schelvis RM, Robroek SJ. Ways to study changes in psychosocial work factors. Scand J Work Environ Health 2023 Mar;49(2):95–8. 10.5271/sjweh.4081 36739541 PMC10577011

[53] McEwen BS. Physiology and neurobiology of stress and adaptation: central role of the brain. Physiol Rev 2007 Jul;87(3):873–904. 10.1152/physrev.00041.2006 17615391

[54] Bobba-Alves N, Juster RP, Picard M. The energetic cost of allostasis and allostatic load. Psychoneuroendocrinology 2022 Dec;146:105951. 10.1016/j.psyneuen.2022.105951 36302295 PMC10082134

[55] Kasl SV. Measuring job stressors and studying the health impact of the work environment: an epidemiologic commentary. J Occup Health Psychol 1998 Oct;3(4):390–401. 10.1037/1076-8998.3.4.390 9805283

[56] Dormann C, Griffin MA. Optimal time lags in panel studies. Psychol Methods 2015 Dec;20(4):489–505. 10.1037/met0000041 26322999

[57] Madsen IE, Rugulies R. Understanding the impact of psychosocial working conditions on workers’ health: we have come a long way, but are we there yet? Scand J Work Environ Health 2021 Oct;47(7):483–7. 10.5271/sjweh.3984 34477882 PMC8504162

[58] Wiegand DM, Chen PY, Hurrell JJ Jr, Jex S, Nakata A, Nigam JA et al. A consensus method for updating psychosocial measures used in NIOSH health hazard evaluations. J Occup Environ Med 2012 Mar;54(3):350–5. 10.1097/JOM.0b013e3182440a04 22382896

[59] Dubrow JK, Tomescu-Dubrow I. The rise of cross-national survey data harmonization in the social sciences: emergence of an interdisciplinary methodological field. Qual Quant 2016;50(4):1449–67. 10.1007/s11135-015-0215-z

[60] Hussong AM, Curran PJ, Bauer DJ. Integrative data analysis in clinical psychology research. Annu Rev Clin Psychol 2013;9:61–89. 10.1146/annurev-clinpsy-050212-185522 23394226 PMC3924786

[61] Singh RK. Harmonizing Instruments with Equating. Harmonization: Newsletter on Survey Data Harmonization in the Social Sciences [Internet] 2020;6(1):11–8. Available from: https://nbn-resolving.org/urn:nbn:de:0168-ssoar-68262-1

[62] Rugulies R, Aust B, Greiner BA, Arensman E, Kawakami N, LaMontagne AD et al. Work-related causes of mental health conditions and interventions for their improvement in workplaces. Lancet 2023 Oct;402(10410):1368–81. 10.1016/S0140-6736(23)00869-3 37838442

